# Start of a new editor-in-chief

**DOI:** 10.1007/s00426-024-02072-2

**Published:** 2025-01-18

**Authors:** Tilo Strobach

**Affiliations:** Medical School Hamburg, Hamburg, Germany

As announced in the last editorial of Bernhard Hommel, he will end his position as Editor-in-Chief by the end of 2024 (Hommel, 2024). He leaves behind a journal after 16 years of a remarkable increase in publication activity. This activity increased from six issues with about 10–14 publications per volume to eight issues with more than 20 publications per volume. There was also a drastic change in publication frameworks during this time. The ever-increasing pace of publication, as well as the increased formalities involved in processing manuscripts during the submission process in order to produce statistics, have made publishing far more formal. These are only a few of the evident changes that have occurred during Bernhard Hommel’s 16-year tenure as Editor-in-Chief of this journal. Furthermore, the journal’s effect in the community as “An International Journal of Perception, Attention, Memory, and Action” has grown, and Psychological Research is recognized as a leading outlet in its field. I think I can speak on behalf of the science community when saying: Thank you, Bernhard, for this great effort.

At this point, it is my honor and pleasure to become the new Editor-in-Chief at the start of 2025. After years of contributing to this journal as an author, reviewer, and associate editor, I will transition to the post of Editor-in-Chief. Of course, this individual transition does not result in any immediate fundamental changes to the journal’s character. My goal is to maintain the journal’s status as a global venue for modern, theoretically directed psychological thinking and experimentation. The journal will also continue to encourage an increased emphasis on theory over methodism. That is, methodological techniques, such as smart experimentation, basically serve as a tool for contributing to theoretical advances in scientific activity. The journal will continue to represent the psychological discipline in its whole, publishing works that are independent of specific schools of thought (Scheerer, 1988) as well as specific societies or interest groups.

Having said that, the journal will continue to expand its focus on open science practices. These practices include ethical developments, methodological and statistical developments, the availability of data and code, author contribution statements, and so on. Furthermore, from 2025 onwards, Psychological Research will overhaul its publication framework. Until this, one-year or longer publication delays were common. This unpleasant process frustrated many people, particularly early-career authors who were eager for their first article to get published, maybe graduate, and pursue a career in academia. However, beginning in 2025, such difficulties will no longer exist. Instead, Psychological Research implements a publication framework with virtually no publication latency. I am sure all authors will appreciate this Continuous Article Production that will help to reduce the latency of publishing. This framework allows papers to be assigned to issues immediately after they are published online. As a result, once the proofing process is completed, the papers are published straight in the current issue. The change in the role of Editor-in-Chief will also be accompanied by a change in the membership of the editorial board. The previous Editor-in-Chief’s term ends, as does the term of the previous board members. This outcome aligns with several members’ future plans, and I am grateful to those members for their time, work, and energy committed to this journal. Other members instead will still serve for the journal and agreed to continue on the next editorial board. I am also grateful to those members for their time, work, and energy, and I am looking forward to continuing to work with them. I also thank all reviewers who spend their efforts in evaluating submissions. Peer review can only work if those who submit their work and expect qualified feedback (authors) are also willing to provide such feedback to others in the role of reviewers. So please engage in reviewing when being asked for, despite the ever-increasing workload of scientific staff, that I am well aware of. The authors submitting to this journal, but eventually the whole system of peer review, depend on this engagement.

Although other developments are not entirely under the control of the journal and its editors, it is envisaged that the journal will evolve in a more diverse manner. For example, in the past, this trend has been evident in terms of the gender distribution and the geographical regions of the authors who publish in Psychological Research, as well as the readers who access these papers. This was accompanied by a larger diversity of the institutions of the authorship and the readership. In a more or less elegant way, this brings me to a more personal note. Although I completed my PhD and some of my academic training at the journal’s founding institution, the Humboldt-Universität zu Berlin (formerly known as the Friedrich-Wilhelms University of Berlin), I am now based at Medical School Hamburg. As a result, I am the first Editor-in-Chief of Psychological Research from a private university, marking a significant novel development in diversifying the European university environment. Furthermore, as far as I can tell, I am the first Editor-in-Chief of Psychological Research to be fully academicized following the unification of West and East Germany and the fall of the Iron Curtain in Europe. This is particularly relevant when considering that there are only a few journals with a lively history and with such a strong imprint from their sociocultural background as Psychological Research. This unique history was remarkably illustrated by a unique set of different phases: the journal’s awakening in the 1920s, its destruction in the 1930s, its comeback after World War II, the consolidation in the 1990s, and its eventual rise in the new millennium (Heuer, 2022). In this way, the journal represents Germany’s upheavals and is a survivor of both political and scientific darkness and light.

I feel honored to be a part of this continuation of the journal’s rich history. This continuation will be societal in nature, but mostly academic, and I hope that our authorship and readership will continue in this vein. My responsibility as Editor-in-Chief will be, as Bernhard Hommel stated (Hommel, 2024), to steer the ship Psychological Research by finding just the right balance between different opposing forces that are shaping the way our discipline brings the scientific news to the world.
